# Comprehensive approach to characterize the circuit between the E and the MVL concerned with visual processing in the avian pallium

**DOI:** 10.1016/j.isci.2026.117356

**Published:** 2026-10-24

**Authors:** Shan Lu, Xiaoteng Zhang, Rui Wang, Yueyang Cang, Yanyan Peng, Xinwei Li, Yingjie Zhu, Li Shi, Shaoju Zeng

**Affiliations:** 1 College of Life Sciences, Beijing Normal University, Beijing, China; 2 Department of Automation, Tsinghua University, Beijing, China; 3 Institute for Brain and Cognitive Sciences, Tsinghua University, Beijing, China; 4 School of Electrical and Information Engineering, Zhengzhou University, Zhengzhou, China; 5 The Brain Cognition and Brain Disease Institute, Shenzhen Institutes of Advanced Technology, Chinese Academy of Sciences, Shenzhen, China

**Keywords:** avian tectofugal visual pathway, neural circuits, SATB2 excitatory neurons, ligand-receptor signaling, single-cell transcriptomics, feedforward/feedback hierarchy

## Abstract

Like most mammals, birds possess advanced visual perception. However, the organization of the avian pallium is largely different from that of the mammalian layered cortex. Here, using viral tracing, the neural connectivity between the entopallium (E) and the mesopallium ventrolaterale (MVL), two visual processing regions in the pallium of the pigeon (Columba livia), was shown to be bidirectional, but dominated by E→MVL. MVL neurons projecting to E or receiving projections from E were SATB2 immunoreactive. According to single-cell RNA sequencing data, NeuronChat indicated that the strength of ligand-receptor interactions in E→MVL was weighted more heavily than that in MVL→E. Electrophysiological studies showed that MVL neurons exhibited more elongated receptive fields. Three-dimensional reconstruction for SATB2 cells in MVL receiving projection from E indicated an enrichment of mature mushroom spines in the proximal dendritic compartment. These results are helpful in deepening the understanding of the neural mechanism underlying visual processing in the avian pallium.

## Highlights

• E ↔ MVL circuit is bidirectional, but dominated by E→MVL
• SATB2 excitatory neurons are distributed in E ↔ MVL circuits
• Strength of ligand-receptor interactions in E→MVL prevails over that in MVL→E
• MVL neurons display elongated receptive fields than E neurons

## Graphical abstract

**Figure:**
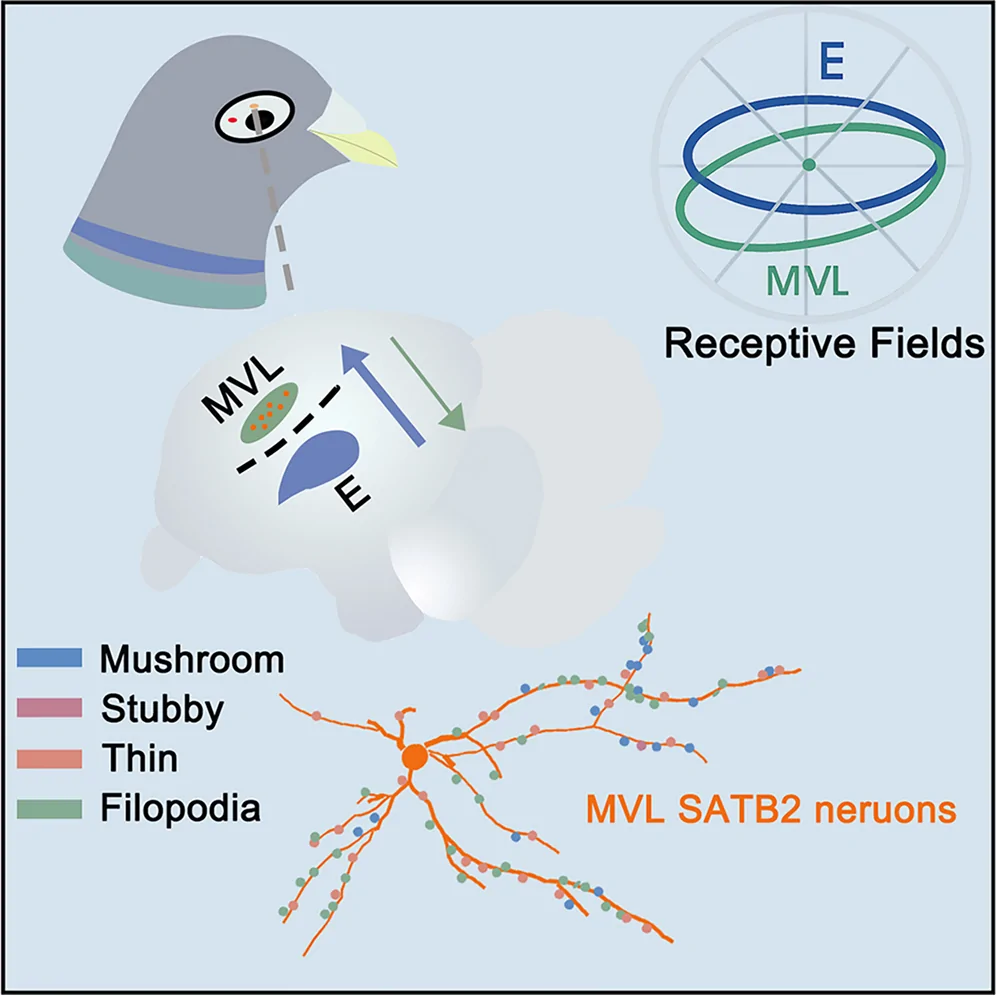

## Teaser

Zoology; Neuroscience; Transcriptomics

## Introduction

Multilayered neural circuits are fundamental for sensory processing and higher-order perception in mammals.^1^^,^^2^^,^^3^^,^^4^ The tectofugal pathway, a sophisticated hierarchical visual system in birds, has been well known, in which the thalamic nucleus rotundus projects to the entopallium (E), and the E projects to further regions of the mesopallium ventrolaterale (MVL), the nidopallium, and the arcopallium.^5^^,^^6^^,^^7^ Of the regions in the tectofugal pathway, the E predominantly processes elementary visual features such as color, orientation, and luminance, and it is regarded as functionally comparable to the mammalian extrastriate cortex.^8^^,^^9^^,^^10^ In contrast, the MVL is involved in higher-level computations, including invariant object recognition and complex category learning.^11^^,^^12^

During amniote evolution, the avian pallium, which is characterized by nuclear organization, has undergone substantial morphological changes from the mammalian neocortex.^13^^,^^14^ Recent comparative developmental and transcriptomic studies indicate that although most inhibitory neuron types are evolutionarily conserved among the amniotes, glutamatergic pallial neurons have diverged.^15^^,^^16^^,^^17^ Studies on the similarities and differences in the neural connectivity of pallial regions, the cytological organization, gene expression, and their physiological functions between mammals and nonmammals are helpful to understand the mechanisms for sensory processing in advanced vertebrates.

SATB2, a homeodomain transcription factor, recruits transcriptional and epigenetic regulators to its DNA-binding sites.^18^ In mammalian embryonic cortex, SATB2 is expressed in superficial cortical layers and regulates the development of callosal/corticocortical projection neuron identity by repressing Ctip2 transcription.^18^^,^^19^^,^^20^ In birds, the mesopallium, including the MVL, has been found to be a region particularly enriched with SATB2.^17^ However, how these SATB2+ neurons are incorporated into the E-MVL circuit for visual processing remains unresolved.

To address the issue, we employed a comprehensive approach to study the E↔MVL circuit. We first applied viral tracing to map the topography of the circuit. Combined with immunocytochemistry staining and reported data on single-cell RNA sequencing in the chick brain,^17^ SATB2 neurons and gene expression were determined in the E-MVL circuit. By using NeuronChat,^21^ ligand-receptor interaction patterns were predicted among neurons in the MVL and E. We then compared the changes in visually evoked receptive fields between the E and MVL. Finally, we studied dendritic spine features of MVL SATB2+ neurons receiving projections from the E. This study provides a comprehensive perspective on the neural circuit underlying visual processing in the avian tectofugal pathway.

## Results

### Pronounced bias of neural connectivity in E↔MVL circuit

To characterize the neural connectivity between the E and MVL, anterograde tracer HSV-H129-4G (herpes simplex virus, titer: 2.00 × 10^9^ PFU/mL) was injected unilaterally into either the E or MVL. To obtain an optimal time for visualizing neuronal projection, HSV labeling was examined at different intervals (36–76 h) after injection (Figure S1A), with reference to previous reports.^22^^,^^23^ At 56 h, HSV labeling was obviously observed in the projecting regions after HSV injection into the E or the MVL, and the 56 h interval was chosen (Figures 1A–1C and S1B–S1D).

**Figure 1:**
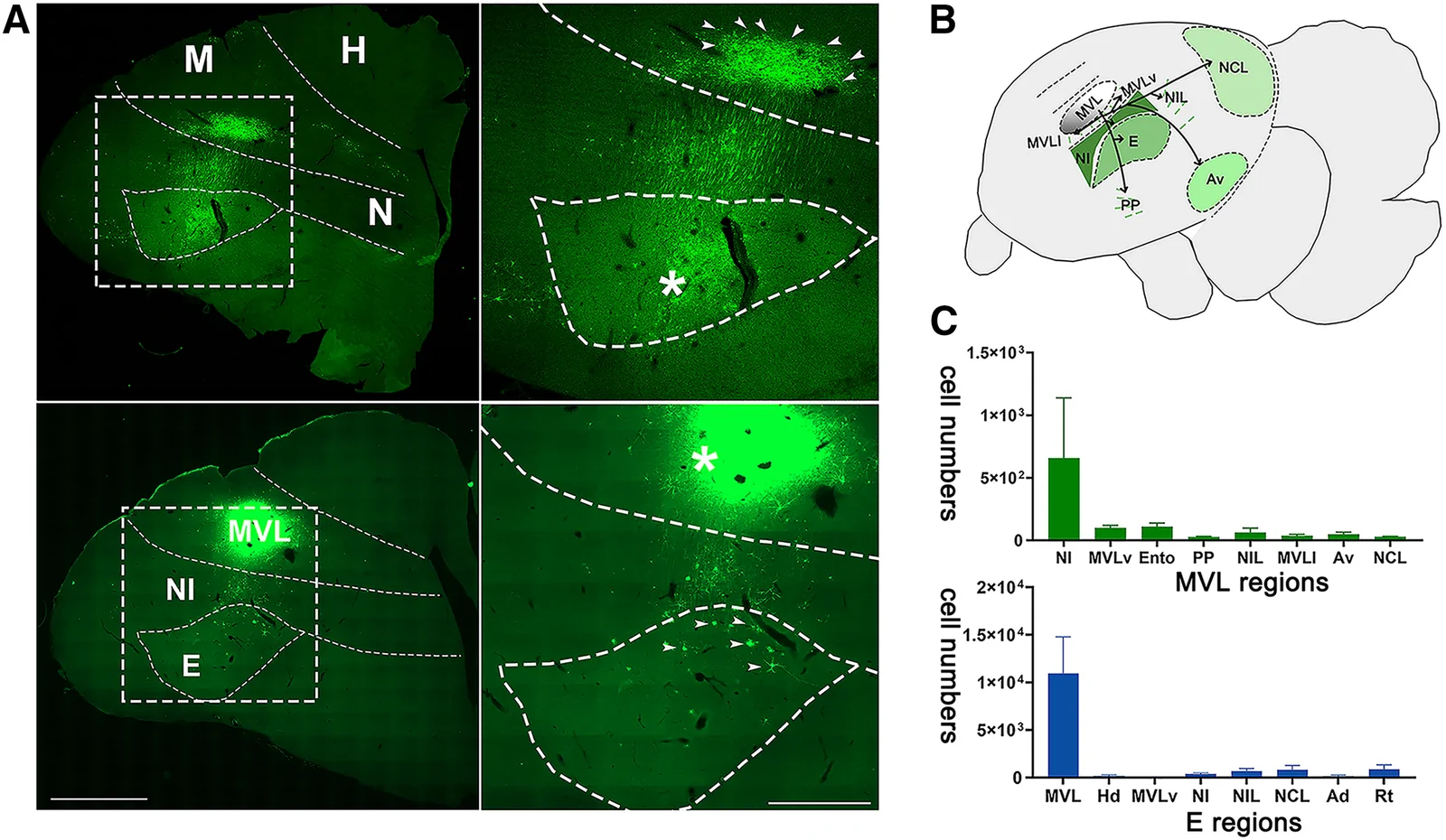
Distinct and region-specific *trans*-synaptic connectivity following HSV-H129-4G injections into the E or MVL (A) Cross-region infection patterns following HSV-H129-4G injection into the E and MVL, showing labeling in the corresponding target regions (*n* = 3 pigeons for each group). Scale bars, 2000 μm and 1000 μm at the right and left bottom of A, respectively. The asterisks indicate injection sites, and the arrows indicate HSV-positive neurons. (B) The distribution of HSV-H129-4G in the whole brain 56 h after viral injection into the MVL (*n* = 3 pigeons for each group). Scale bars, 2000 μm. (C) Quantification of HSV-labeled neurons across brain regions at 56 h after viral injection into the E (top, blue) and MVL (bottom, green). Data represent the mean ± SEM (*n* = 3 pigeons for each group). For the abbreviations, see the list in the text. MVL = Mesopallium ventrolaterale, H = Hyperpallium, M = Mesopallium, N = Nidopallium, Hd = Densocellular part of hyperpallium, NIL = Nidopallium intermediate pars lateralis, NCL = Nidopallium caudolaterale, PP = Paleostriatum primitivum, Rt = Nucleus rotundus, Ad = Dorsal arcopallium, Av = Acropallium pars ventralis, and MVLv = Ventral MV.

After HSV injection into the MVL, labeled cells were sparsely distributed in the E, whereas dense labeling appeared within the MVL after HSV injection into the E (Figures 1A, S2, and S3), indicating a pronounced bias of neural connectivity between the E and the MVL.

In addition, some double-labeled neurons were observed in the MVL after the injections of anterograde HSV and retrograde CTB-555 (Cholera toxin subunit B) into the same site of E. This indicates that the same neurons in the MVL receive afferent innervation from the E and, in return, send output feedback to the E, forming a close E↔MVL circuit (Figure 2A).

**Figure 2:**
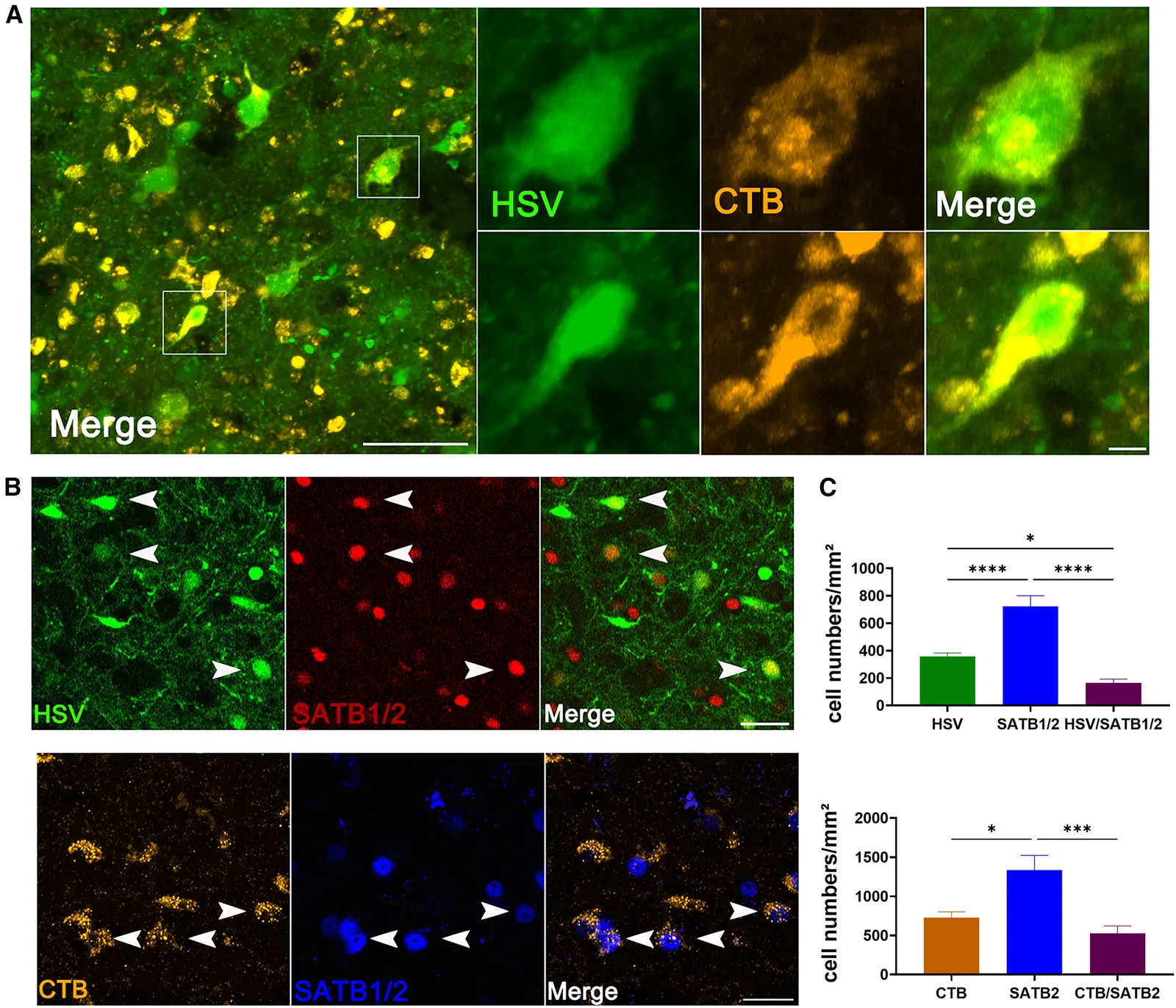
SATB1/2-expressing neurons in the E-MVL pathway (A) Co-labeling of MVL neurons following the dual injection of CTB and HSV into the same site of the internal part of the entopallium (*n* = 3 pigeons). Scale bars, 50 μm. (B) Representative fluorescence images that show SATB1/2 expression in MVL neurons that are labeled with CTB and HSV injections into the E. Scale bars, 20 μm. (C) Quantification of SATB1/2-positive neuron densities among retrogradely labeled MVL neurons shown in (B), following CTB (*n* = 7 pigeons) or HSV (*n* = 9 pigeons) injection into the E. Data are presented as mean ± SEM. Statistical significance was assessed using one-way ANOVA. One-way ANOVA revealed significant differences among the three groups in HSV-labeled neurons (HSV, SATB1/2, and HSV/SATB1/2 neurons; *F* = 32.32, *p* < 0.0001) and among the three groups in CTB-labeled neurons (CTB, SATB1/2, and CTB/SATB1/2 neurons; *F* = 10.44, *p* < 0.001). ∗*p* < 0.05, ∗∗*p* < 0.01, and ∗∗∗*p* < 0.001; ns, not significant. HSV one-way ANOVA *F* = 32.32, *p* < 0.0001; CTB one-way ANOVA *F* = 10.44, *p* < 0.001.

### Immunocytochemistry for SATB1/2 and gene expression in the E and MVL

Given the marked structural asymmetry within the E↔MVL loop, we next sought to determine whether SATB2 subtypes were present in the circuit. As shown in Figures 2B and 2C, immunocytochemistry staining for SATB1/2 indicated that some retrogradely or anterogradely labeled MVL neurons, following CTB or HSV-H129-4G into the E, respectively, were also positively labeled. We have noted single-cell transcriptomic results in the chick brain from Zaremba et al.,^17^ indicating that among the excitatory neurons in the region corresponding to the MVL shown in a stereotaxic atlas of the brain of the chick (Gallus domesticus),^24^ the majority of cells were either SATB2-positive only or double-positive for SATB1 and SATB2, and there was only a small proportion of cells expressing SATB1 alone (Figures S4 and S5). Thus, although both SATB1 and SATB2 can be recognized by the antibody used in the present immunostaining according to the protocol, the double-labeled cells obtained above were predominantly for SATB2 and CTB or HSV labeling.

Referred to a previous report on transcriptomic analysis in the chick brain,^17^ we determined gene expression of neurons in the MVL and E. The positions and sizes of MVL and E were determined based on a stereotaxic atlas of the brain of the chick (Gallus domesticus),^24^ and the region of interest (ROI) masks for the MVL and E were manually delineated in the brain for transcriptomic analysis^17^ (Figure 3A, more details on the determination of the MVL and E are shown in STAR Methods). A total of 75 cells in the MVL and 1,620 cells in the E were obtained for further transcriptomic analysis. The results revealed that the E was significantly enriched for the excitatory subtypes Ex_DACH2_ADAMTS5 and Ex_DACH2_ZMAT4 (adjusted *p* = 0.016 for both, Fisher’s exact test with BH correction), whereas the MVL was highly enriched for Ex_SATB2_ZNF385B and Ex_KIAA1217 excitatory subtypes (adjusted *p* = 1.03 × 10^−10^ and 7.27 × 10^−6^, Fisher’s exact test with BH correction) (Figures 3A, 3B, S4B, and S6A).

**Figure 3:**
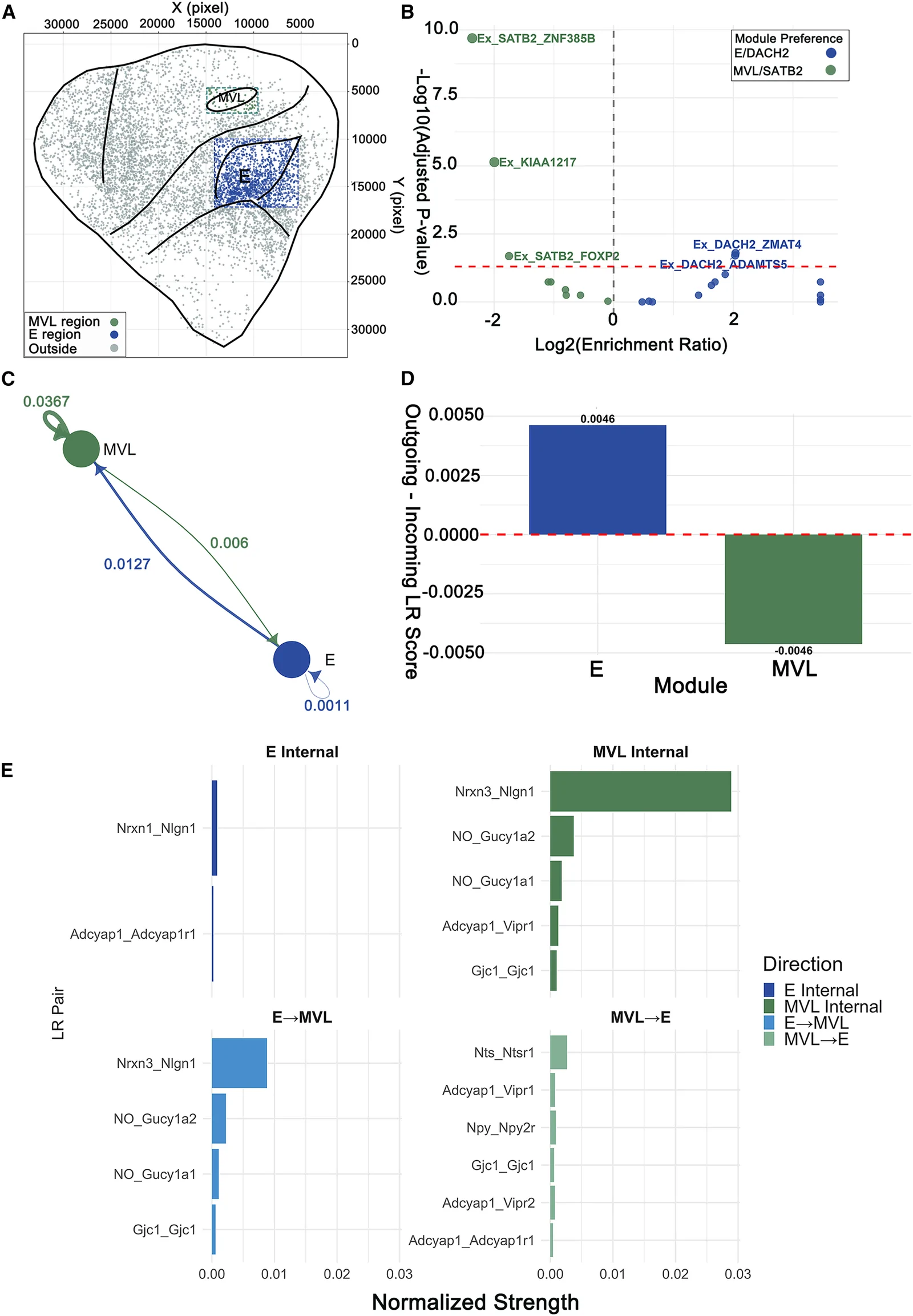
Spatial distribution of cells for transcriptomic analysis and ligand-receptor communication between the entopallium (E) and the mesopallium ventrolaterale (MVL) (A) Spatial distribution of cells performed for transcriptomic analysis based on physical coordinates, rotated 180° counterclockwise for visualization. The two green dashed boxes indicate the regions of interest (ROIs) used for MVL and E delineation. The outlines of MVL and E are determined based on a stereotaxic atlas of the brain of the chick (Gallus domesticus).^24^ (B) Volcano plot showing cell-type enrichment between the E and MVL modules. The X axis represents the log_2_ enrichment ratio, and the Y axis represents the –log_10_ adjusted *p* value. Significantly enriched cell types (FDR < 0.05) are labeled. (C) Circular plots illustrating predicted directional ligand-receptor communication networks between the E and MVL modules, based on transcriptome-derived interaction potentials. (D)Outgoing- Incoming Score for each module. Positive values indicate net sender modules. (E) Top 10 ligand-receptor pairs across four predicted directionalities: E internal, MVL internal, E→MVL, and MVL→E. Bars represent normalized interaction strength.

### NeuronChat analysis indicated ligand-receptor interactions in E ↔ MVL circuit

Using NeuronChat, we next examined ligand-receptor interaction patterns among neuronal subtypes in the MVL and E (Figure S6A). To link this dataset with the spatial annotations, we performed a spatial enrichment analysis, then mapped these module identities back to the dataset to enable NeuronChat analysis (More details in STAR Methods).

Enriched ligand-receptor interactions were predicted within the MVL, particularly those concerned with adhesion-related pairs such as Nrxn3–Nlgn1 and Nrxn1–Nlgn1 (Figure 3A). Directional inference based on aggregated ligand-receptor interaction weights suggested a bias toward E→MVL interactions, with predicted E→MVL interaction strength exceeding MVL→E by a factor of 2.12. This asymmetry is consistent with the anatomical bias observed in the viral tracing experiment (Figure 3C). The cross-module interaction density (0.0046) was higher than the internal interaction, suggesting a pronounced inter-regional molecular interaction potential (Figure 3D).

Ex_DACH2 neurons in the E were predicted to show preferential ligand-receptor interaction potential with MVL neurons, particularly via adhesion-related pairs including Nrxn3–Nlgn1 and Nrxn1–Nlgn1 (Figure 3E). There were predicted E→MVL interactions by gaseous and peptidergic signaling, including NO-Gucy1a1 and Adcyap1-Vipr1 pairs. In contrast, inferred MVL→E interactions were relatively sparse and dominated by Nts-Ntsr1 (Figure 3E).

### Receptive field geometry varied between the E and MVL

To determine how the identified anatomical and molecular specializations are concerned with physiological responses to visual stimuli, we compared the changes in visually evoked receptive fields between the E and MVL (RFs; Figures 4A, 4C, and 4D). Although RF areas did not differ significantly between the E and MVL (E: *n* = 16, 332.87 ± 43.37 px^2^; MVL: *n* = 24, 338.93 ± 50.88 px^2^; independent samples *t* test, *t* = 0.390, *p* = 0.698), MVL -RFs exhibited significantly higher aspect ratios than E-RFs (2.31 vs. 1.90; Mann-Whitney *U* test, *p* = 0.006), forming more elongated elliptical MVL-RFs (Figure 4D). In addition, the E and MVL both showed a strong concentration of RF orientations toward the N-NE direction (0°–48°). However, MVL-RFs showed significantly reduced angular dispersion of RF orientation axes, with a markedly lower circular standard deviation in contrast to that of E-RFs (4.24° vs. 14.16°, Rayleigh test, *Z* = 30.10(E), *Z* = 47.74(MVL), both *p* < 0.001; Figures 4C and 4D), indicating a more concentrated orientation preference toward the N-NE direction in the MVL (mean angle: 13.70° vs. 16.60°).

**Figure 4:**
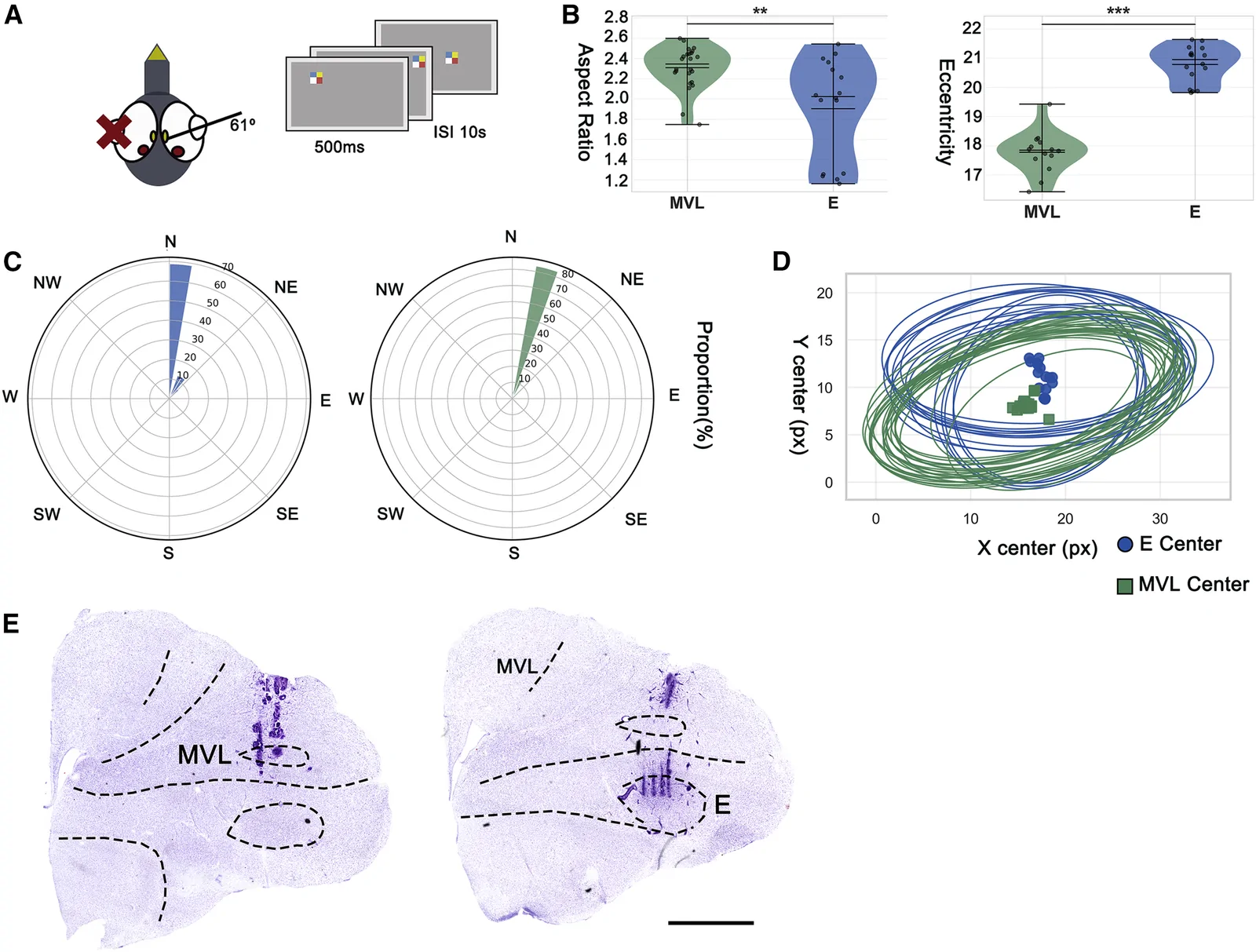
Receptive field (RF) for the recording neurons' properties and direction tuning in the entopallium (E) and the mesopallium ventrolaterale (MVL) (A) Schematic illustration of the visual stimulation setup. A 3.6° colored square stimulus was displayed for 500 ms at random positions within the visual field (at a distance of 20 cm and an angle of 30° from the body axis). (B) Violin plots that compare key receptive field parameters between E and MVL neurons: Aspect ratio (Mann-Whitney *U* test, *p* = 0.005), and Eccentricity (independent samples *t* test, *p* < 0.001). Data are represented as mean ± SEM. *n* = 16 neurons from 2 pigeons for E; *n* = 24 neurons from 2 pigeons for MVL. ∗*p* < 0.05, ∗∗*p* < 0.01, and ∗∗∗*p* < 0.001. (C) Polar histograms of RF orientation distribution in the E and MVL, showing the angular distribution of preferred RF orientation axes for E (left, blue) and MVL (right, green) neurons. The polar angle represents the preferred RF orientation axis (θ), defined as the direction of the major axis of the two-dimensional Gaussian-fitted RF boundary, with 0° corresponding to North (N), 90° to East (E), 180° to South (S), and 270° to West (W). Bar height (radius) indicates the proportion of cells (%). Both regions exhibit a strong concentration of RF orientations toward the N-NE direction (0°–48°). Angular dispersion, quantified by circular standard deviation, is lower in the MVL (*σ* = 4.33°) than in the E (*σ* = 14.62°). *n* = 16 neurons from 2 pigeons for E; *n* = 24 neurons from 2 pigeons for MVL. (D) Spatial distribution and morphological properties of receptive fields in the E and MVL. Overlay of RF boundaries from E (blue ellipses) and MVL (green ellipses) neurons, with each ellipse representing the RF boundary fitted by a two-dimensional Gaussian function. Blue circles and green squares denote the RF centers of E and MVL neurons, respectively. The X and Y axes represent the spatial coordinates of the RF center in pixels (px). *n* = 16 neurons from 2 pigeons for E; *n* = 24 neurons from 2 pigeons for MVL. (E) Electrode placement in the MVL or E is shown. Scale bars, 2000 μm.

### Three-dimensional reconstruction of SATB2+ projection neurons in the MVL reveals dendritic spine features

To reveal dendritic spine features, we quantified the spatial distribution of dendritic spines in MVL SATB2+ neurons receiving projections from the E. The spatial distribution of distinct dendritic spines was three-dimensionally reconstructed for SATB2+ neurons in the MVL labeled by HSV injections into the E (Figures 5A–5D). Using Neurolucida with four threshold parameters (e.g., head-to-neck ratio and spine length; see STAR Methods for the other parameters), dendritic spines were automatically classified into four types: stubby, mushroom, thin, and filopodia. Across reconstructed neurons (*n* = 14), the mean total dendritic tree length was 524.4 ± 133.4 μm, and the spine density was 6.27 ± 0.71 per 10 μm (Figure 5E). Sholl analysis revealed dendritic arbors extending to 106.4 ± 12.1 μm from the soma, with peak branching complexity at 30.7 ± 4.1 μm (Figure 5F).

**Figure 5:**
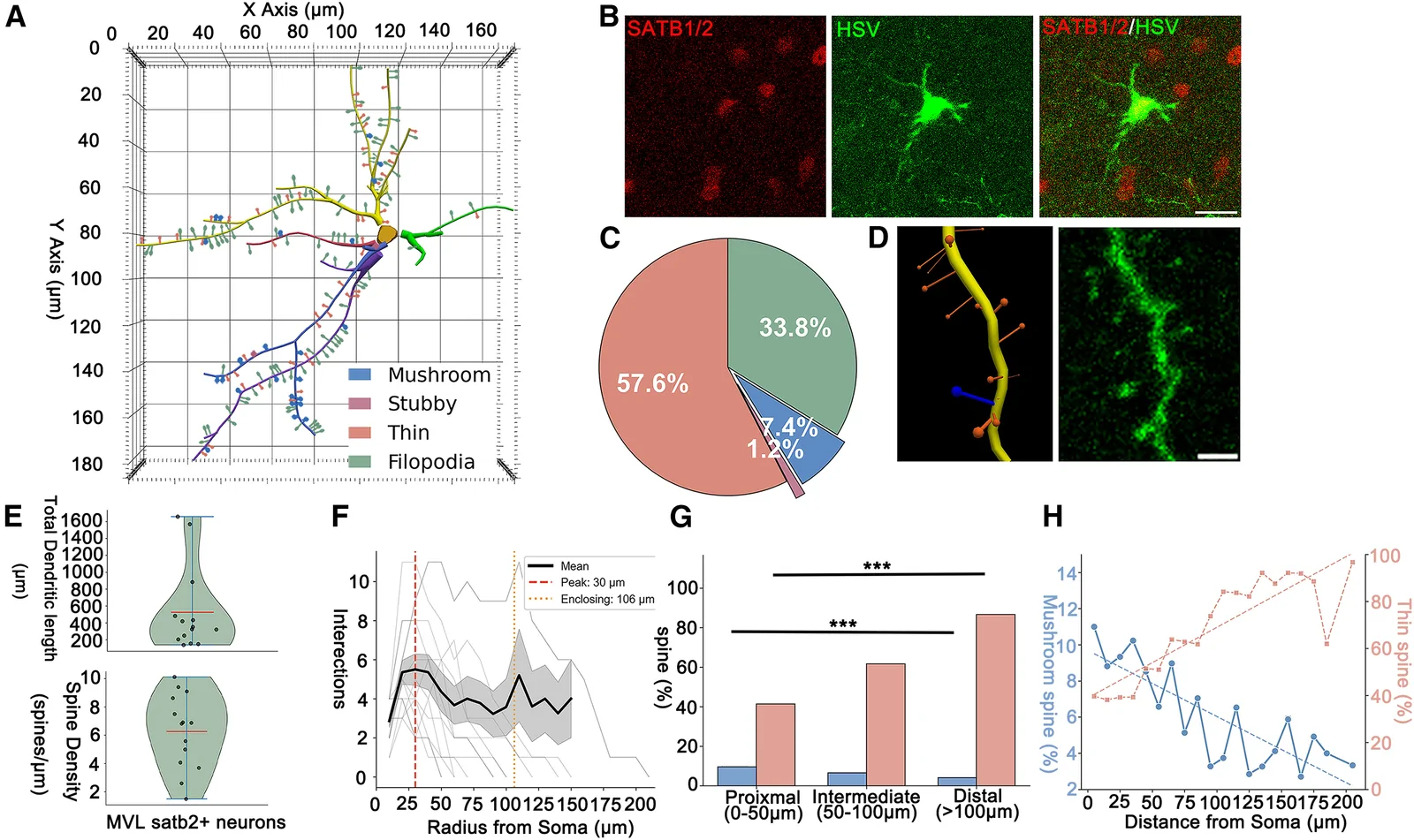
Dendritic spines of SATB2+ neurons in the mesopallium ventrolaterale (MVL) receiving projections from the entopallium (E) (A) Three-dimensional reconstruction of a representative SATB2+ neuron in the MVL, showing the spatial distribution of distinct dendritic spine types (mushroom, stubby, thin, and filopodia) from an oblique perspective. Note that the spines are manually enlarged using Photoshop based on their locations; therefore, the spine sizes in the image are not to scale and should not be used as a reference. (B) Fluorescence images show a SATB2+ neuron in the MVL corresponding to the reconstructed neuron in (A), following HSV tracing from the E. The neuron is co-labeled with SATB1/2 (red) and HSV (green). Scale bars, 20 μm. (C) Pie chart illustrates the composition of dendritic spine types, including thin, mushroom, and stubby spines. (D) Reconstruction of dendritic spines on a representative dendritic segment using Neurolucida 360. Subtype assignment is color-coded according to the same scheme as in (D). Scale bars, 2 μm. (E) Total dendritic length (top, mean ± SEM: 524.4 ± 133.4 μm) and dendritic complexity index (bottom, 3873.3 ± 1517.7) for individual MVL SATB2+ neurons (*n* = 14 neurons from 3 pigeons). Each point represents one neuron. (F) Sholl analysis curves show the number of dendritic intersections at increasing distances from the soma, with mean ± SEM highlighted. (G) Quantification of mushroom and thin spine distribution along the proximal-distal axis. Dendritic compartments are classified based on Sholl analysis (proximal, 0–50 μm, peak complexity zone; intermediate, 50–100 μm, transition zone; distal, >100 μm, beyond enclosing radius). Mushroom spines (blue bars) decrease, but thin spines (coral bars) increase from the proximal to the distal compartment. All pairwise comparisons between the compartments are statistically significant (Mann-Whitney *U* test, *p* < 0.001, Bonferroni-corrected). Values are shown as percentages ±SEM. ∗∗∗*p* < 0.001 for proximal vs. distal comparison. (H) Spatial distribution of mushroom and thin spines along the proximal-distal axis. Dendritic compartments were defined by Sholl analysis. Values are mean ± SEM (*n* = 14 neurons from 3 pigeons).

Using a Sholl-based sampling method,^25^ a dendritic path was divided into three compartments based on Sholl curves: proximal (0–50 μm), intermediate (50–100 μm), and distal (>100–328 μm) (Figure 5G). The results indicated that mushroom spines were preferentially enriched in the proximal compartment, whereas thin spines progressively accumulated in the distal compartment (mushroom: proximal 9.5% to distal 4.0%, Kruskal-Wallis H = 35.62, *p* < 0.0001; linear regression slope = −0.37%/10 μm, *R*^2^ = 0.672, *p* < 0.0001; thin: proximal 41.3% to distal 86.7%, Kruskal-Wallis H = 647.15, *p* < 0.0001; linear regression slope = +3.01%/10 μm, *R*^2^ = 0.763, *p* < 0.0001; Figures 5G and 5H).

## Discussion

This research characterizes the E-MVL circuit in the avian tectofugal pathway from neural connectivity, neuron type, ligand-receptor interaction, receptive field geometry, and dendritic spine features. At the level of neural connectivity, time-resolved viral tracing revealed pronounced asymmetry in the E↔MVL circuit: E→MVL projections were substantially more numerous, whereas MVL→E projections were relatively sparse. Thus, this projection pattern is characterized by a numerically dominant feedforward and a low-density feedback, which is consistent with the pattern reported previously.^26^^,^^27^ It is necessary to point out that the anatomical location and the size of MVL are difficult to determine in Nissl-stained brain sections. However, this problem can be overcome by using cytochrome oxidase staining and retrograde CTB-labeling after CTB injection into the E (Figure S1). The methods were thus used to help determine the location and the size of MVL during HSV or CTB injection, and during analysis of labeling in the MVL. In addition, all the projection results obtained by HSV were consistent with the reports using other neural tract tracers.^5^^,^^6^^,^^7^

Some double-labeled neurons were observed in the MVL after the injections of anterograde HSV and retrograde CTB-555 into the same site of E, forming a close neural circuit between the E and MVL. Our immunocytochemistry study further indicated that some retrograde projection neurons from the E and anterograde projection neurons to the E were both SATB2+ neurons. The projection patterns provide a substrate for visual processing in which broad, distributed inputs from the E could converge onto the MVL, whereas the sparse MVL→E projections may have a more selective influence on the E. However, more studies on the physiological functions of the circuit between the E and MVL, especially the closure circuit formed by SATB2+ neurons, are needed.

With reference to the study on single-cell RNA sequencing,^17^ the analysis revealed that the MVL was highly enriched for excitatory subtype Ex_SATB2_ZNF385B and Ex_KIAA1217 subtypes, whereas the E was significantly enriched for excitatory subtype Ex_DACH2_ADAMTS5 and Ex_DACH2_ZMAT4. Using NeuronChat analysis, enriched ligand-receptor interaction potential was predicted within the MVL, including adhesion-related pairs and gaseous and peptidergic signaling. Given the established roles of neurexin-neuroligin signaling in synapse organization and maturation across cortical circuits,^28^^,^^29^ *trans*-synaptic adhesion may contribute to the specificity and stabilization of E-derived inputs onto MVL. In addition, regarding the fact that nitric oxide-cGMP signaling is related to activity-dependent synaptic modulation,^30^ the predicted neuromodulatory interactions may influence the gain and integration of E-derived inputs in MVL neurons. Finally, there was a bias toward E→MVL interactions, with predicted E→MVL interaction strength exceeding MVL→E, which was consistent with the anatomical bias observed in the viral tracing experiment.

Electrophysiological study showed that MVL neurons exhibited significantly higher RF aspect ratios, markedly reduced dispersion of RF orientation axes, and a more strongly elongated and population-aligned anisotropic RF structure, in contrast to E neurons. Such features have been regarded as being concerned with effective coupling between spatial frequency and orientation processing by reducing variability in RF orientation.^31^^,^^32^^,^^33^

Our study further indicated that there were distinct spine types along the spatial segregation of dendrites in MVL SATB2+ neurons receiving projections from the E. There was a pronounced enrichment of mushroom spines within the proximal segment, and thin spines in the distal compartment. These mushroom spines may be involved in processing relatively stable, locally weighted inputs, as suggested by a previous report on the compartmentalized dendritic organization of synaptic inputs *in vivo*.^34^ In contrast, the plastic thin spines have been shown to facilitate local functional clustering of synaptic inputs and dendritic nonlinear integration.^35^

### Limitations of the study

Using a comprehensive analysis of the expression patterns of SATB1 and SATB2, including the published single-cell transcriptomic data, we are confident that the immunohistochemical signals observed in the MVL are predominantly derived from SATB2. Nevertheless, we acknowledge that this analysis cannot fully rule out the possibility of nonspecific antibody binding.

Based on transcriptomic data on the chick brain,^17^ NeuronChat analysis predicted a bias of ligand-receptor interactions toward E→MVL, which was consistent with the anatomical bias observed in viral tracing experiments. However, it is necessary to note that the NeuronChat ligand-receptor database was developed for mouse and human datasets.^21^ Despite stringent one-to-one ortholog filtering, cross-species application remains outside its primary scope and carries inherent limitations, including potential species-specific differences such as post-translational modifications that may influence ligand-receptor function. In addition, although the comparisons of large-scale data from single-cell RNA sequencing of neurons were performed cross-species (from the songbird, chick, turtle and mouse),^15^^,^^16^^,^^17^ and these comparisons did provide us with some useful knowledge, the cross-species strategy may introduce additional uncertainty.^36^ Thus, the obtained conclusions should be interpreted with caution.

It is needed to point out that unlike GABAergic lineages of neurons, glutamatergic pallial neurons, including SATB2-expressing populations, are not strongly conserved between birds and mammals on the basis of comparative developmental and transcriptomic studies.^19^^,^^20^^,^^36^^,^^37^ It calls for caution against direct comparisons of SATB2-associated circuit functions obtained in this study with those reported in mammals. Nevertheless, SATB2 is used here primarily as a lineage or organizational marker, rather than implying conserved mammalian circuit function.

In addition, 3D reconstructions of MVL SATB2+ neurons were obtained from brain slices with a thickness of approximately 50 μm. During the slicing process, parts of neuronal dendrites may have been truncated. This incomplete structural preservation could lead to an underestimation of morphological features, such as total dendritic length and branching complexity.

## Resource availability

### Lead contact

Requests for further information and resources should be directed to and will be fulfilled by the lead contact, Shaoju Zeng (sjzeng@bnu.edu.cn).

### Materials availability

This study did not generate new unique reagents.

### Data and code availability

• The 3D reconstruction HSV-neuron ASC files, NeuronChat-processed outputs, and visual field analysis data supporting this study have been deposited at Zenodo and are publicly available as of the date of publication at https://doi.org/10.5281/zenodo.18374032.
• All original code has been deposited at Zenodo and is publicly available at https://doi.org/10.5281/zenodo.18374031 as of the date of publication. The code is also maintained at https://github.com/luxima98/avian-tectofugal-satb2-gain-control.
• Single-cell transcriptomic data and *in situ* hybridization data analyzed in this paper were obtained from a publicly available dataset (https://doi.org/10.11588/data/BX6REK).
• Any additional information required to reanalyze the data reported in this paper is available from the lead contact upon request.

## Acknowledgments

This work was supported by the 10.13039/501100001809National Natural Science Foundation of China (grant no 32470493 to Shaoju Zeng). We thank the members of the Zeng laboratory for their helpful discussions and technical support. We thank the Beijing Key Laboratory of Gene Resources and Molecular Development, College of Life Sciences, Beijing Normal University, for providing experimental facilities.

## Author contributions

S.L., X.Z., Y.Z., and L.S. conceived the idea of this project. S.L. developed a surgical process and performed all the pigeon experiments, with the assistance of Y.P. and S.L. processed and analyzed the immunohistochemistry data. X.Z. processed and analyzed the electrophysiological data. X.Z. developed the neural network model simulating the Ento-MVL connection, with the assistance of Y.C. L.S., Y.Z., and S.Z. supervised all aspects of the project. S.L. and X.Z. made figures and wrote the manuscript with feedback from all the authors, supported by the assistance of X.L., R.W., and S.Z. Y.Z. revised and polished the paper.

## Declaration of interests

The authors declare no competing interests.

## Declaration of generative AI and AI-assisted technologies in the manuscript preparation process

During the preparation of this work, the authors used AI-assisted tools, including the NeuronChat pipeline and electrophysiology visualization, in order to assist with code development. After using these tools, the authors reviewed, edited, and manually validated all AI-generated code as needed, confirmed that no AI tools were used to generate or alter research data or conclusions, and took full responsibility for the integrity of the manuscript and the content of the publication.

## STAR★Methods

### Key resources table

**Table:** 

REAGENT or RESOURCE	SOURCE	IDENTIFIER
**Antibodies**		

Anti-Satb1/2 primary antibody	Abcam	PRID: ab51502^4^
Alexa Fluor 594-conjugated donkey anti-mouse antibody	Abcam	PRID: AB150013^5^
Alexa Fluor 405-conjugated donkey anti-mouse antibody	Abcam	PRID: ab175658^6^

**Bacterial and virus strains**		

HSV-H129-4G	Genechem (Shanghai, China)	>2 E+9 PFU/mL
CTB-555	BrainVTA (Wuhan, China)	10 μg/ul

**Biological samples**		

*Pigeons (Columba livia)*	Mingxue Pigeon Farm (Hebei, China)	Female or male

**Chemicals, peptides, and recombinant proteins**		

Donkey Serum	YTHX Biotechnology (Beijing, China)	CAT#SL050
Triton ×100	Sigma	T9284
Urethane	aladdin	U299635
4% paraformaldehyde (PFA)	YTHX Biotechnology (Beijing, China)	CAT#HF004

**Critical commercial assays**		

Cytochrome Oxidase Stain Kit (DAB Method)	Solarbio	Cat#G2410

**Deposited data**		

Adult chicken pallial single-nucleus RNA sequencing (snRNA seq) dataset	Zaremba et al.^17^	https://doi.org/10.11588/data/BX6REK
Adlut chicken *in situ* sequencing (ISS) data	Zaremba et al.^17^	https://doi.org/10.11588/data/BX6REK

**Software and algorithms**		

ZEN 3.0 (Blue Edition)	ZEISS	3.0
ZEN 2009 (Light Edition)	ZEISS	2009
Fiji	ImageJ	https://fiji.sc/
GraphPad Prism 9	GraphPad Software	Version 9
MATLAB	MathWorks	R2025.a
MATLAB Psychophysics Toolbox	MathWorks^38^	Psychtoolbox-3
Apollo System	Blackrock Microsystems	Apollo System
Kilosort	Pachitariu et al.^39^	NA
Neurolucida 360	MBF Bioscience	2020.1
Seurat	Hao et al.^40^	v5.0.0
biomaRt	Bioconductor	biomaRt R package
SCTransform	Hafemeister et al.^41^	v0.4.3
NeuronChat	Zhao et al.^21^	v1.0.0
Baysor	Petukhov et al.^42^	Python package
Tangram	Biancalani et al.^43^	Python package
pandas	The pandas development team^44^	v3.0.2
Numpy	Harris et al.^45^	v2.4.0
Scipy	Virtanen et al.^46^	v1.17.0
scikit-learn	Scikit-learn developers^47^	v1.8.0
seaborn	Waskom, M.^48^	v0.13.2
scales	Wickham et al.^49^	v1.2.1
plotly	Plotly Technologies Inc.	https://plotly.com
ggplot2	Wickham, H.^50^	v3.4.0
ggalluvial	Brunson, J.C.^51^	NA
ComplexHeatmap	Gu et al.^52^	v2.14.0
igraph	igraph developers^53^	v2.1.4
Scanpy	Wolf et al.^54^	Python package
Gseapy	Fang et al.^55^	Python package
RColorBrewer	Neuwirth, E.^56^	v1.1.3

**Other**		

LCD monitor	NA	3840 × 2160 pixels
ZEISS Confocal Laser Scanning Microscope	ZEISS	LSM880
ZEISS inverted fluorescence microscopy	ZEISS	Observer Z1
Cryostat Microtome	Lecia	CM3050s
Apollo Portable Neural Recording System	BIo-Signal	NA
16-channel silicon probes	kedoubc	custom

4 Repalced by: PRID:ab51502

5 Repalced by: PRID:ab51502

6 Replaced by PRID:ab175658

### Experimental model and study participant details

#### Experimental animals

Forty adult male and female pigeons (Columba livia; 350–450 g) were used in this study. All animals were acclimated for at least two weeks before experimentation in temperature- and humidity-controlled rooms under a natural light–dark cycle, with *ad libitum* access to food and water. To ensure experimental independence, the pigeons were singly housed following surgery. All animal procedures and tissue processing were conducted in accordance with the institutional guidelines of Beijing Normal University and were approved by the Institutional Animal Care and Use Committee (IACUC) of Beijing Normal University (approval number: CLS-AWEC-B-2023–004).

### Method details

#### Stereotaxic surgeries and viral injections

Stereotaxic surgeries were performed under deep anesthesia that was induced by 20% urethane (0.3 mL/100 g). Target regions were localized according to the pigeon brain atlas using the distance between the bregma and the ear bars as a reference. The stereotaxic coordinates were as follows^57^:

MVL: AP +11.0, ML 4.5, DV 2.0 mm; Entopallium (E): AP +10.75, ML 5.3, DV 4.7 mm.

Neural tracers or viral vectors—including the retrograde transsynaptic tracer including the anterograde transsynaptic tracer HSV-H129-4G (Genechem, Shanghai, China; titer: 2.00 × 10^9^ PFU/mL; derived from HSV-1 strain H129, carrying an mGFP-2A-eGFP cassette under the CMV promoter; all HSV work conducted in a BSL-2 facility) and the retrograde tracer CTB-555 were slowly delivered using a microsyringe, with each injection site receiving 200 μL. To prevent reflux and ensure complete absorption, the injection needle was left in place for 5–10 min before withdrawal. The following infection intervals were established according to neural tracers or viral vectors: 46, 56, or 72 h for HSV and 7 days for CTB. For the double-labeling experiments, HSV injection into the internal entopallium (Ei) was performed 7 days after CTB delivery into the same hemisphere.

#### Histology, immunofluorescence, and morphological analysis

Following the tracer or viral incubation period, the pigeons were deeply anesthetized and perfused transcardially with saline followed by 4% paraformaldehyde (PFA). Their brains were postfixed in 4% PFA and cryoprotected in 30% sucrose. Coronal sections were cut at 50 μm for immunofluorescence.

#### Immunofluorescence

The sections were blocked in 5% donkey or goat serum (YTHX Biotechnology, Beijing, China) that was supplemented with 0.5% Triton X-100 and incubated overnight at 4 °C with mouse anti-Satb1/Satb2 primary antibody (Abcam, ab51502; 1:100). After being washed with PBS, the sections were incubated at room temperature for 2 h with the following Alexa Fluor 594-conjugated secondary antibodies: Alexa Fluor 594-conjugated donkey anti-mouse antibody (1:500; Abcam AB150013) and Alexa Fluor 405-conjugated donkey anti-mouse antibody (1:100; Abcam ab175658). The sections were mounted with antifade medium.

#### Imaging

Whole-brain fluorescence images were acquired using a ZEISS inverted fluorescence microscope (20× objective), and high-resolution images from specific regions were obtained with a ZEISS LSM700 confocal microscope (63× oil objective) using z stack acquisition. Image processing was performed in ZEN 3.0 and ZEN 2009, and cell counting was conducted in Fiji.

#### Quantification of SATB1/2+ neurons and CTB/HSV-labeled in the MVL

For each animal (*n* = 9), the MVL region was identifiable in approximately four consecutive coronal sections. The two sections with the largest MVL cross-sectional area were selected for analysis (∼50% sampling fraction). In each section, two ROIs were delineated bilaterally (left and right), flanking the core MVL labeling area. For each ROI, a single optical plane was selected at the focal depth providing the sharpest nuclear profile discrimination and minimal cellular overlap. Double-labeled neurons were defined by co-localization of both signals within the same cell profile at the counted optical plane. Neuron counts were normalized by ROI area to calculate density (neurons/mm^2^), and values were averaged across ROIs and sections to yield a single data point per animal. All counting was performed by an investigator blinded to experimental group.

#### Single-cell transcriptomics and spatial transcriptomics

##### Single-cell processing and cross-species gene mapping

Single-cell transcriptomic data were obtained from a publicly available dataset^17^ (https://doi.org/10.11588/data/BX6REK). Quality control and normalization were performed using Seurat v5.0.0. To improve the robustness of NeuronChat inference, rare cell types (<50 cells) were iteratively merged (for up to three rounds) on the basis of Euclidean distances in the variable gene space.

Cross-species gene mapping was achieved using OrthoFinder-based orthology tables to convert chicken Ensembl IDs to mouse Ensembl IDs, which was followed by conversion to mouse gene symbols with biomaRt. A weighted scoring system (orth_score:seq_score:domain_score:expr_score = 0.4:0.2:0.2:0.2) was applied to resolve many-to-many orthologous relationships (Figures S7 and S8) The expression matrices were normalized using SCTransform while regressing out technical confounders.

##### Spatial identification of transcriptomic cells in the E and MVL

Spatial transcriptomic data were converted into AnnData objects and segmented using Baysor. To integrate spatial and single-cell transcriptomic information, Tangram was trained for 500 epochs to map scRNA-seq cell-type profiles onto the spatial transcriptomic dataset. For each spatial location, cell-type assignments were determined according to Tangram mapping probabilities, and only the top 75% highest-confidence assignments were retained for downstream analyses.

The positions and sizes of MVL and E in the chick brain performed transcriptomic analysis^17^ were determined based on a stereotaxic atlas of the brain of chick (*Gallus domesticus*).^24^ The region of interest (ROI) masks for the MVL and E were thus manually delineated in Fiji on the reference spatial image, with the MVL spanning X = 9623.56–14927.45 and Y = 4636.71–7223.29, and E spanning X = 5332.51–14084.83 and Y = 10021.28–17129.22. Segmented cells were assigned to the MVL, E, or outside both regions of both the E and MVL according to their centroid coordinates, yielding 75 cells in the MVL and 1,620 cells in the E.

Cell-type enrichment within the E or MVL regions was quantified by comparing the observed abundance of each cell type against the spatial background distribution. Cell types were classified as E-enriched or MVL-enriched ones according to the enrichment statistics and abundance thresholds. Specifically, cell types satisfying P_value adj_ < 0.05, E_enrichment_ > 1, and E_count_ ≥ 10 were assigned to the E module, whereas cell types satisfying P_value adj_ < 0.05, E_enrichment_ < −1, and MVL_count_ ≥ 10 were assigned to the MVL module. These spatially derived module annotations were subsequently mapped back to the single-cell transcriptomic dataset for the analysis of ligand–receptor interaction network analyses.

##### Ligand–receptor communication analysis

Cell–cell communication was inferred from the single-cell transcriptomic dataset using NeuronChat v1.0.0 with the mouse ligand–receptor database. Communication significance was evaluated using permutation testing with a false discovery rate threshold of 0.05. Cross-species confidence was calibrated using orthology and sequence conservation scores.

After NeuronChat inference, spatially defined E-module and MVL-module labels were mapped onto the corresponding cell types in the single-cell dataset. All significant ligand–receptor (LR) interactions were then aggregated at the module level to generate a 2 × 2 communication matrix M, where each element M_ij_ represents the total communication weight from sender module i to receiver module

j, summed across all significant LR pairs:

$$M_{ij}=\sum_{p\in significant}w_{p}\left(i\to j\right)$$

Where *i*,*j*∈{*E*,*MVL*}, and *w*_*p*_(*i*→*j*) denotes the NeuronChat weight of LR pair p from module i to module j. The four elements are thus M_EE_(E internal), M_EM_(E→MVL), M_ME_(MVL→E), and M_MM_(MVL internal).

To account for differences in module size, raw communication weights were normalized by cell abundance. For intra-module connections (M_EE_ and M_MM_), the normalized weight was calculated per 1,000 cells of the sender (also receiver) module:

$$w_{norm}^{intra}=\frac{w_{raw}}{C_{module}}\times 1000$$

For inter-module connections (M_EM_ and M_ME_), to control for unequal sampling between the two modules, the normalized weight was calculated using the geometric mean of the sender and receiver cell counts:

$$w_{norm}^{inter}=\frac{w_{raw}}{\sqrt{C_{E}\cdot C_{MVL}}}\times 1000$$

From the aggregated network, we derived several module-level metrics. Active LR Pair Density was defined as the proportion of database LR pairs that passed significance testing for a given connection type. Outgoing (S^out^) and incoming (S^in^) communication scores were calculated by summing all sender-associated or receiver-associated communication strengths, respectively. For example, for module E:

$$S_{E}^{out}=M_{EE}+M_{EM},S_{E}^{in}=M_{EE}+M_{ME}$$

$$S_{MVL}^{out}=M_{MM}+M_{ME},S_{MVL}^{in}=M_{MM}+M_{EM}$$

Directional asymmetry was then quantified as the difference between outgoing and incoming strengths:

$$\Delta_{E}=S_{E}^{out}-S_{E}^{in}=M_{EM}-M_{ME}$$

To enable direct comparison between modules with different cell abundances, we further normalized the outgoing and incoming scores per 1,000 cells of the target module:

$${Score}_{E}^{out}=\frac{M_{EE}+M_{EM}}{C_{E}}\times 1000$$

$${Score}_{E}^{in}=\frac{M_{EE}+M_{ME}}{C_{E}}\times 1000$$

$${Score}_{MVL}^{out}=\frac{M_{MM}+M_{ME}}{C_{MVL}}\times 1000$$

$${Score}_{MVL}^{in}=\frac{M_{MM}+M_{EM}}{C_{MVL}}\times 1000$$

#### Electrophysiological recording and analysis

##### Signal acquisition and visual stimulation

Neural activity was recorded using a 16-channel Apollo acquisition system at a 30 kHz sampling rate. Extracellular signals were recorded using commercial 16-channel silicon probes (kedoubc, 4 × 4 grid arrangement, 200 μm inter-channel spacing). For receptive field mapping and direction selectivity assessment, an E-probe (10 mm shank length) was used; for MVL analysis, an MVL-probe (3 mm shank length) was used. Visual stimuli were presented to the right eye via a high-resolution LCD monitor (3840 × 2160 pixels with a 100 Hz refresh rate) while the left eye was completely occluded with a blackout mask. The display was positioned 20 cm from the right eye^58^ and fixed at a 29° angle relative to the midline, which covered 138° horizontally and 112° vertically.

Stimuli were generated using the MATLAB Psychophysics Toolbox. For receptive field (RF) mapping, 3.6° patches were randomly presented across the visual field for 500 ms with 10-s interstimulus intervals.

##### Electrophysiological recording and spike sorting

Raw electrophysiological signals were acquired from two animals in the E and three animals in the MVL. Signals from all sessions for each animal were concatenated and aligned according to synchronized timestamps. High-frequency spike signals were extracted using a 300–3000 Hz bandpass filter. Spike sorting was performed using Kilosort, followed by manual curation in Phy. Units were included only if they exhibited clear refractory periods in the autocorrelogram.

##### Quality control

Units were retained based on the following criteria: (1) ISI violations <1% within a 2-ms window; (2) stable waveform shape and amplitude across recording halves (cross-correlation >0.85 between halves); (3) waveform similarity across spikes within a unit; (4) autocorrelogram structure consistent with single-unit activity; (5) cluster separation quantified using feature-space isolation metrics; and (6) physiological firing rates between 0.5 and 50 Hz. Units with atypical waveforms were excluded. After quality control, 101 well-isolated single units were retained. Multiple neurons were recorded from the same animals (E: 2 animals; MVL: 3 animals), and neurons were treated as independent observations without animal-level random effects.

##### Tuning and receptive field analysis

Spike times were aligned with stimulus timestamps, and 84 data points surrounding each spike were extracted to compute the mean spike waveform. For receptive field (RF) spatial analysis, ellipse-fitting was applied to quantify area, eccentricity, center location, and orientation.

For orientation selectivity, a doubly-angle transformation was applied to account for axial symmetry (0° ≡ 180°), and concentration was quantified using the Rayleigh R statistic. Analyses assumed a predominantly unimodal angular distribution; no formal bimodality test was performed.

##### Statistical analysis

Data normality was assessed using the Shapiro–Wilk test, and variance homogeneity was assessed using Levene’s test. For comparisons of eccentricity and RF area, normally distributed data were analyzed using Welch’s *t* test when variances were unequal; for non-normally distributed data (e.g., aspect ratio), the Mann–Whitney U test was applied.

##### Three-dimensional reconstruction of SATB2+ projection neurons in the MVL

Neuronal reconstruction of HSV+/SATB2+ neurons in the MVL projecting from the E was performed using Neurolucida 360 (2020.1). Dendrites were traced manually, whereas the soma and spines were reconstructed using the software’s automatic detection functions. All parameter settings were recorded for reproducibility. Dendritic spines were automatically classified using Neurolucida with threshold parameters set to a head-to-neck ratio of 1.1, a length-to-head ratio of 2.5, a mushroom head size of 0.35 μm, and a filopodium length of 3 μm.

Using Neurolucida Explorer, we extracted raw measurements including total dendritic length (μm), total surface area (μm^2^), total volume (μm^3^), number of primary dendrites (trees), total branch points (nodes), and terminal endings. Then, sholl analysis was performed to quantify the spatial distribution of dendritic branching complexity as a function of radial distance from the soma. For each neuron, we computed: (1) peak radius, the radial distance from the soma at which the number of dendritic intersections was maximal; (2) peak intersections, the maximum intersection count; (3) enclosing radius, the maximum radial extent of the dendritic arbor; and (4) Sholl AUC, the area under the intersection-versus-radius curve, calculated using trapezoidal integration to quantify overall dendritic sampling capacity.

To examine the spatial organization of spine types along the dendritic axis, we utilized individual spine data that included spine type classification and dendritic path distance from the soma (μm). Dendritic compartments were defined based on Sholl analysis as previously described^26^: proximal (0–50 μm, corresponding to the peak complexity zone), intermediate (50–100 μm, transition zone), and distal (>100–328 μm, beyond the enclosing radius). Within each compartment, the percentage of each spine type was calculated as the proportion of total spines in that compartment belonging to mushroom or thin subtypes. Between-compartment differences in spine type percentages were assessed using the Kruskal-Wallis H test (non-parametric one-way ANOVA), followed by post-hoc pairwise comparisons using the Mann-Whitney U test with Bonferroni correction for multiple comparisons. Post-hoc characterization of spatial gradients was performed using linear regression to quantify the rate of change in spine type percentage per unit distance (slope, expressed as %/10 μm), with the coefficient of determination (R^2^) reported as a measure of fit. All data are presented as mean ± SEM.

### Quantification and statistical analysis

For all experiments, the numbers of biological replicates are indicated in the corresponding figure legends. The independent sample sizes for neuronal tracing, immunofluorescence, and neuronal reconstruction experiments met the minimum requirement of three biological replicates. For electrophysiological experiments, the sample size for each brain region was two independent biological samples.

Data are presented as mean ± standard error of the mean (SEM). Statistical analyses were performed using GraphPad Prism software (v9). Comparisons among multiple groups were performed using one-way analysis of variance (one-way ANOVA), as indicated in the corresponding figure legends. For electrophysiological experiments involving comparisons between two groups, an unpaired two-tailed Student’s *t* test was used. Statistical analysis of three-dimensional neuronal reconstruction data was performed using the Kruskal–Wallis test.

All statistical details, including exact sample sizes (n), definitions of biological replicates, statistical tests, and *p* values, are provided in the corresponding figure legends and/or the results section. Statistical significance was defined as ∗*p* < 0.05, ∗∗*p* < 0.01, ∗∗∗*p* < 0.001, ∗∗∗∗*p* < 0.0001, and ns (not significant).
