# Supplementary material for: Comprehensive approach to characterize the circuit between the E and the MVL concerned with visual processing in the avian pallium
Source: iScience. 2026 Oct 24;29(11):117356. doi: 10.1016/j.isci.2026.117356 (PMC13720825; doi:10.1016/j.isci.2026.117356)
Supplement: Supplementary file 1 [file mmc1.pdf]

iScience, Volume *29*

Supplemental information

Comprehensive approach to characterize the circuit between the E and the MVL concerned with visual processing in the avian pallium

Shan Lu, Xiaoteng Zhang, Rui Wang, Yueyang Cang, Yanyan Peng, Xinwei Li, Yingjie Zhu, Li Shi, and Shaoju Zeng

Supplemental information

Document S1. Figures S1–S8

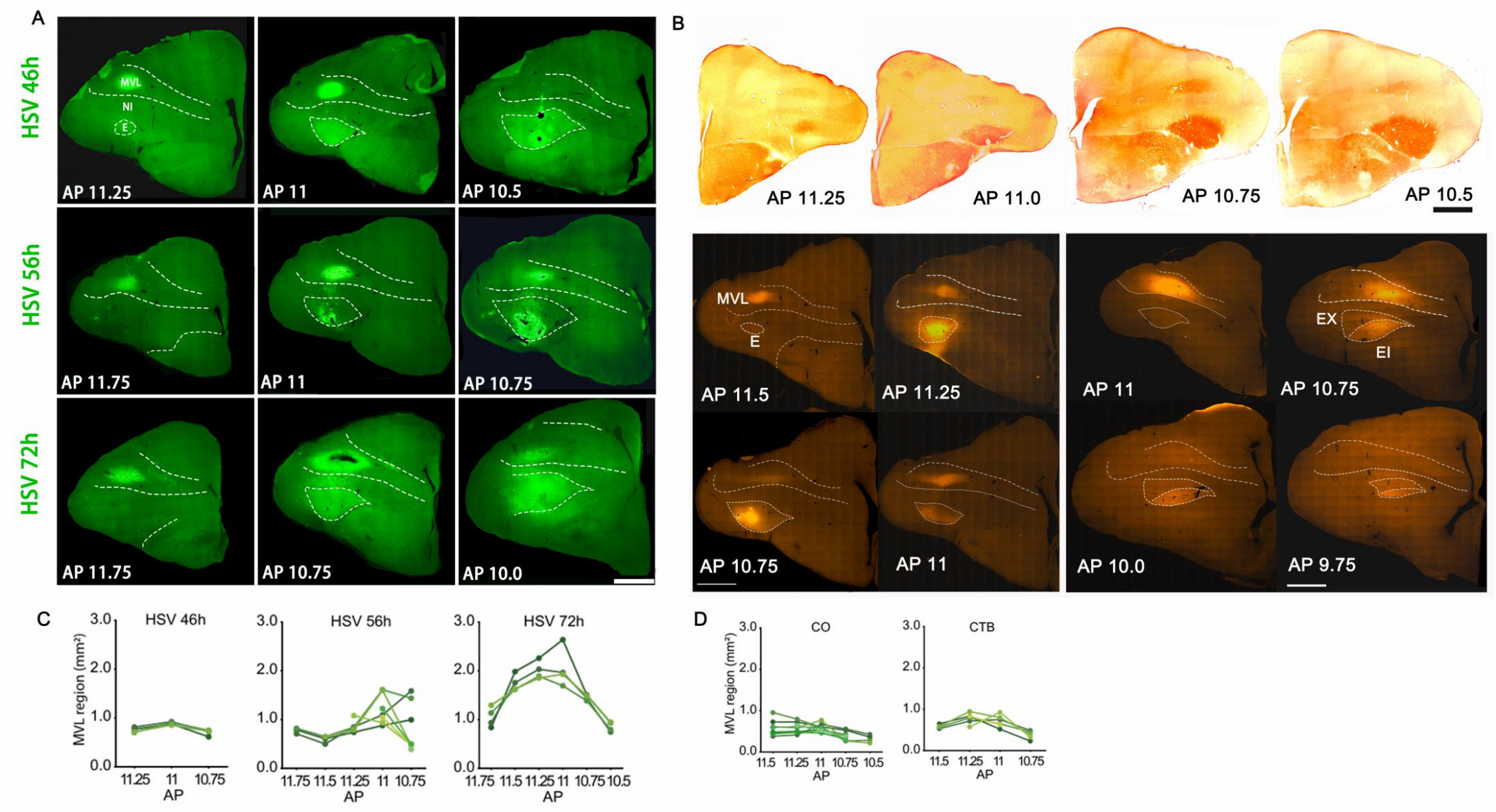

Figure S1. Delineation of the mesopallium ventrolaterale (MVL) based on HSV/CTB labeling

Related to Figure 1.

(A) Distributions of HSV infected neurons in the MVL at 46 h, 56 h, and 72 h following the injection into the internal entopallium (E). Scale bar, 2000 μm.

(B) Identification of MVL boundaries using cytochrome oxidase staining (top) and retrograde CTB labeling (bottom left). Subdivisions of E were labeled 7 days after CTB injection into the MVL (bottom right). Scale bar, 2000 μm.

(C) Quantification of the labeled MVL area (mm²) at three HSV infection time points (n = 3, pigeons).

(D) Comparison of MVL areas determined by cytochrome oxidase staining, CTB labeling, and HSV tracing (n = 4, pigeons). For the abbreviations, see the list in the text.

Abbreviations: AP= Anteroposterior, MVL= Mesopallium ventrolaterale, EI= Internal entopallium, EX= External entopallium.

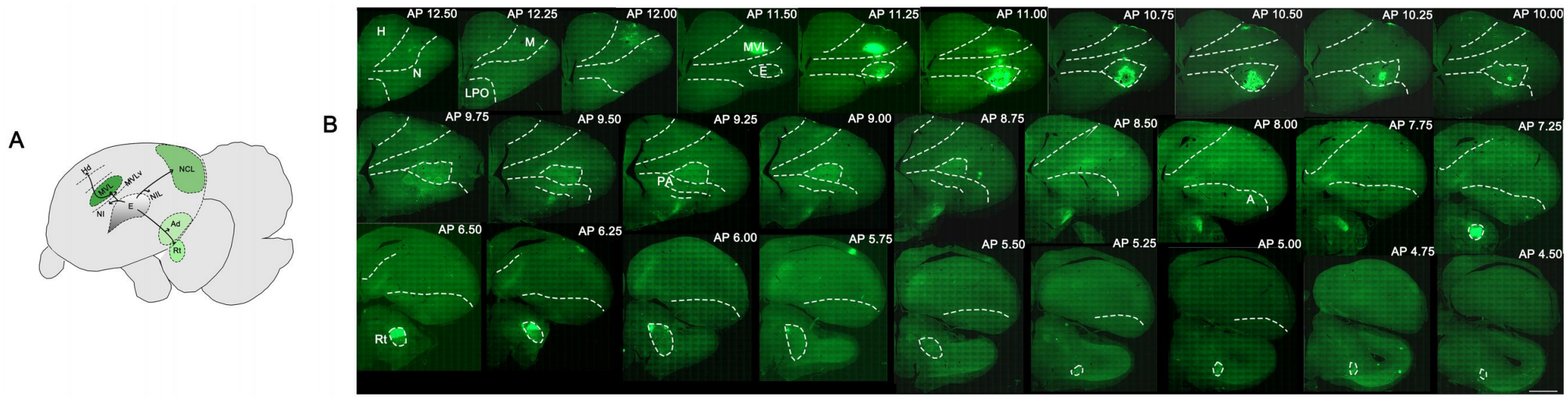

Figure S2. Labeling in the MVL after HSV injection into the E

Related to Figure 1.

(A) Labeling distribution in the brain 56 h after HSV-H129-4G injection into the E (n = 3, pigeons). Scale bar, 2000 μm.

(B) Labeled regions in the brain 56 h after HSV-H129-4G injection into the E. For the abbreviations, see the list in the text.

Abbreviations: AP= Anteroposterior, H= Hyperpallium, NI= Nidopallium, Hd= Den socellular part of hyperpallium, NIL= Nidopallium intermediate pars lateralis, NC L= Nidopallium caudolaterale, Rt= Nucleus rotundus, Ad= Dorsal arcopallium, MVLv = Ventral MV, MVL= Mesopallium ventrolaterale, EI= Internal entopalliu m, EX= External entopallium.

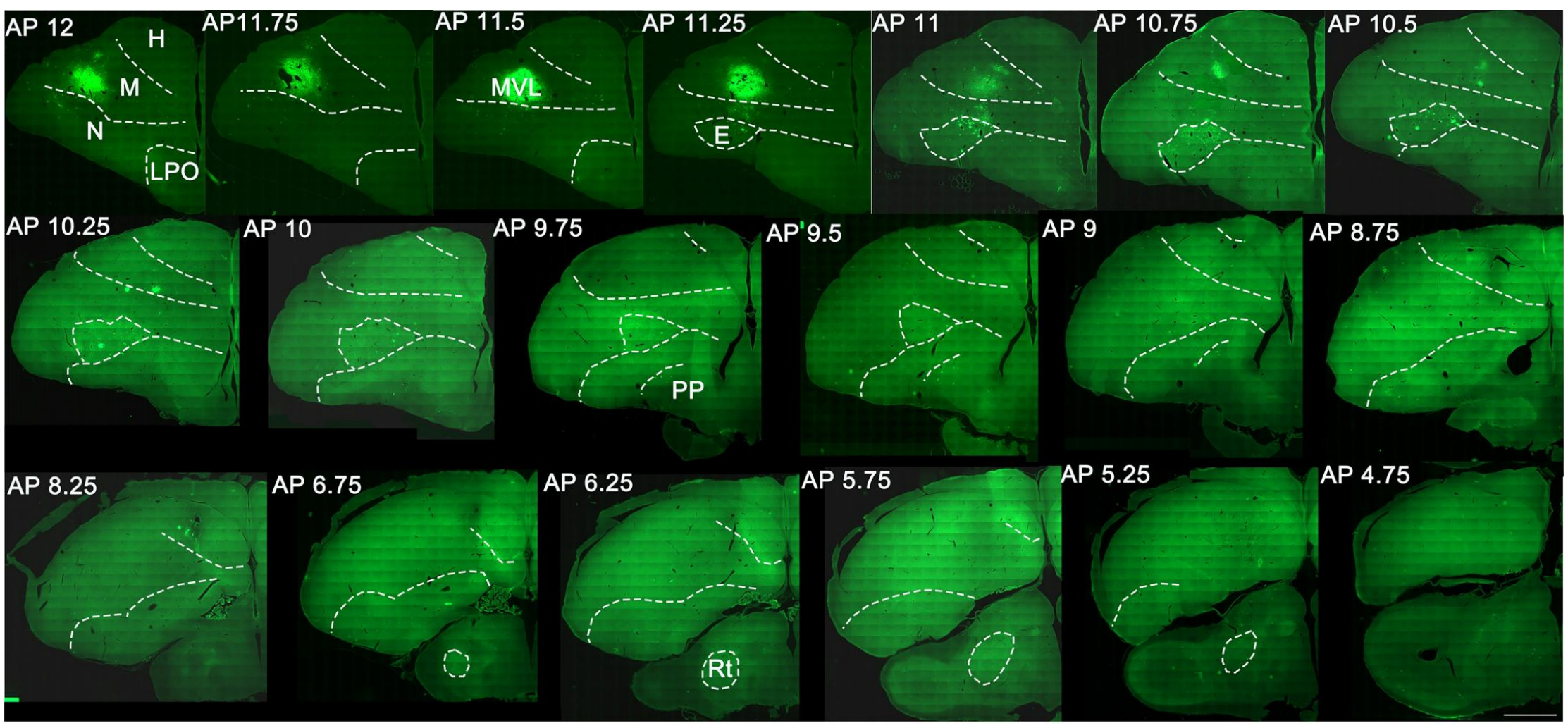

Figure S3. Labeling in the E after HSV injection into the MVL

Related to Figure 1.

Scale bar, 2000 μm. Abbreviation: LPO= Lobus parolfactorius.

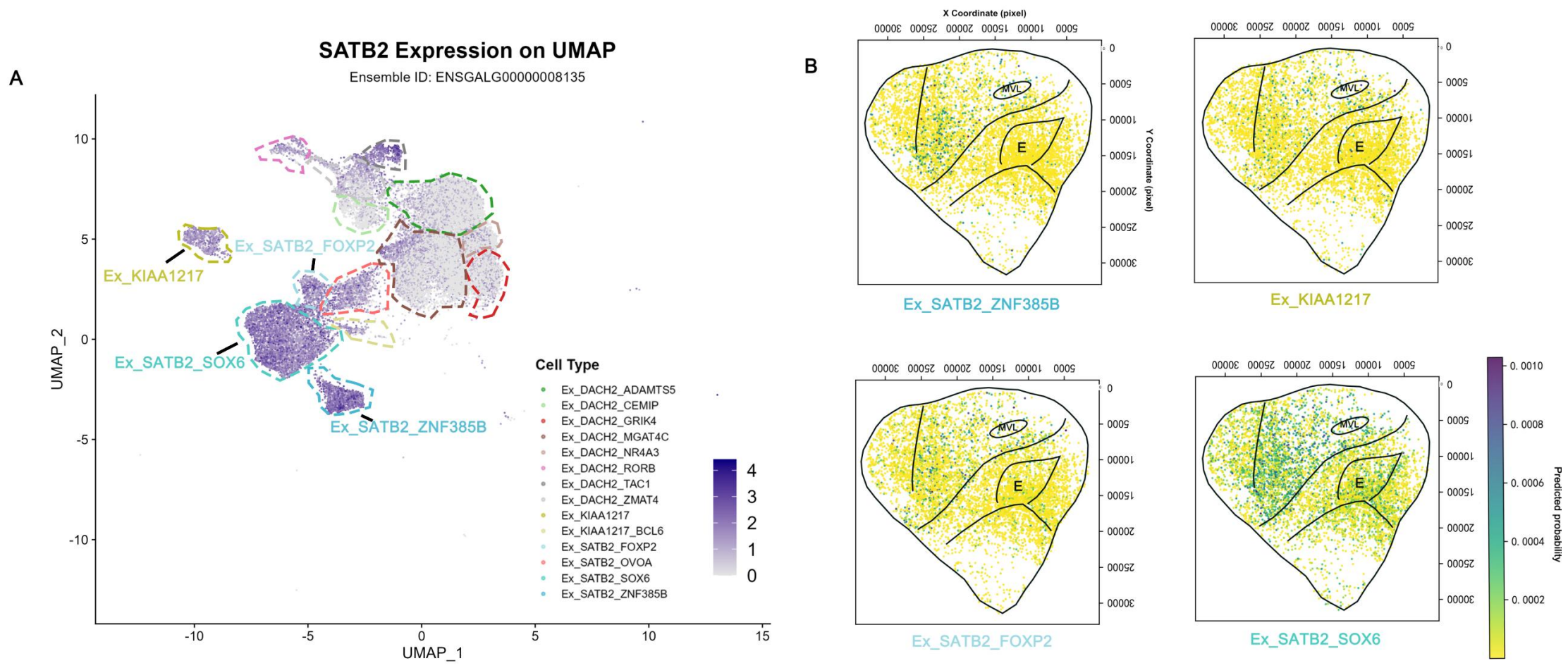

Figure S4. Satb2 population in the MVL and its spatial distribution in the pallium of the chick

Related to Figure 2, 3.

(A) Satb2 expression on UMAP.[17]

(B) Spatial distribution of Satb2 cell types in the pallium of the chick.[17] The positions and sizes of MVL and E are determined based according to a stereotaxic atlas of the brain of chick (*Gallus domesticus*)[24]

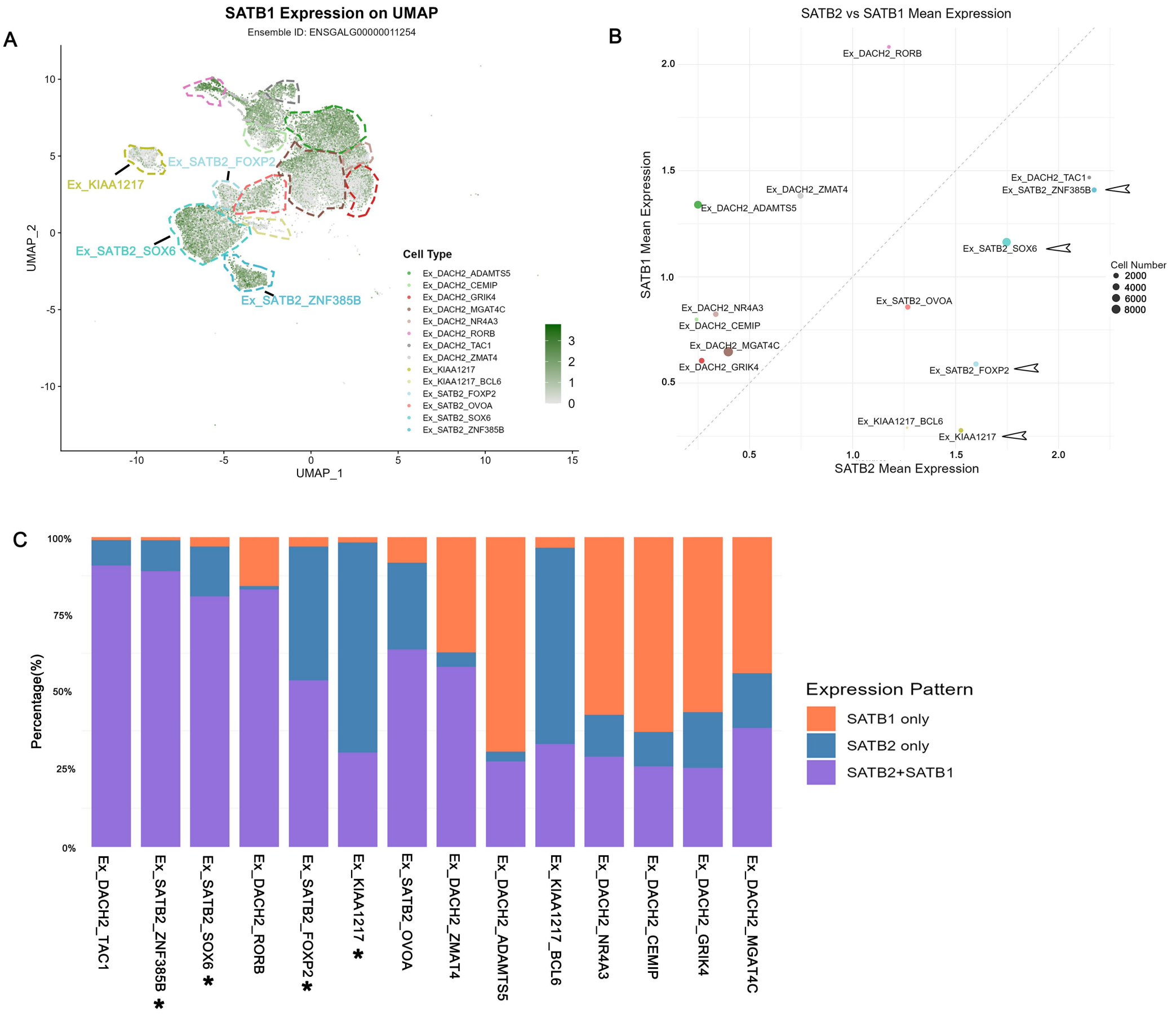

Figure S5. Differential expression patterns of SATB1 and SATB2 cell types in the MVL

Related to Figure 2.

(A) Satb1 expression on UMAP.[17]

(B) Scatter plots showing the average expression levels of Satb2 and Satb1 cell types. Arrows indicate MVL neuronal cell types.

(C) Stacked bar plots illustrating the proportions of four SATB expression patterns (SATB2-only, SATB1-only, double-positive, and double-negative). Asterisks denote MVL neuronal populations.

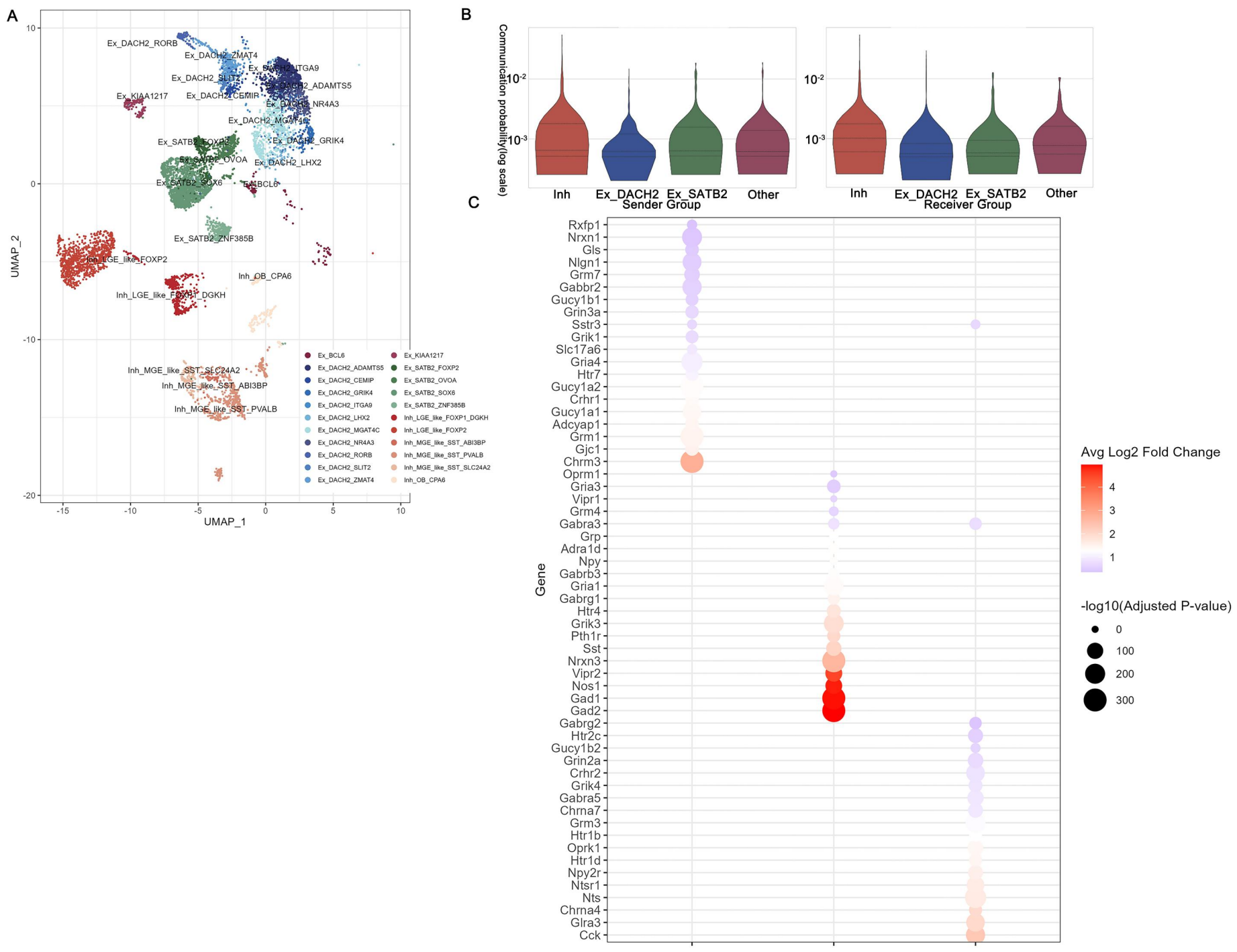

Figure S6. Single-cell transcriptomic analysis of cell-type clusters and ligand–receptor interactions in the mesopallium and nidopallium

Related to Figure 3.

(A) UMAP visualization of cell-type clusters in the mesopallium and nidopallium based on single-cell transcriptomic data.[17]

(B) Violin plots showing the distribution of communication probabilities grouped by sender (top) and receiver (bottom) cell type. The Y-axis is displayed on a log scale.

(C) Dot plots of significantly upregulated ligand–receptor-related genes across neuronal groups. Color: mean log2-fold change; dot size: –log10 (FDR).

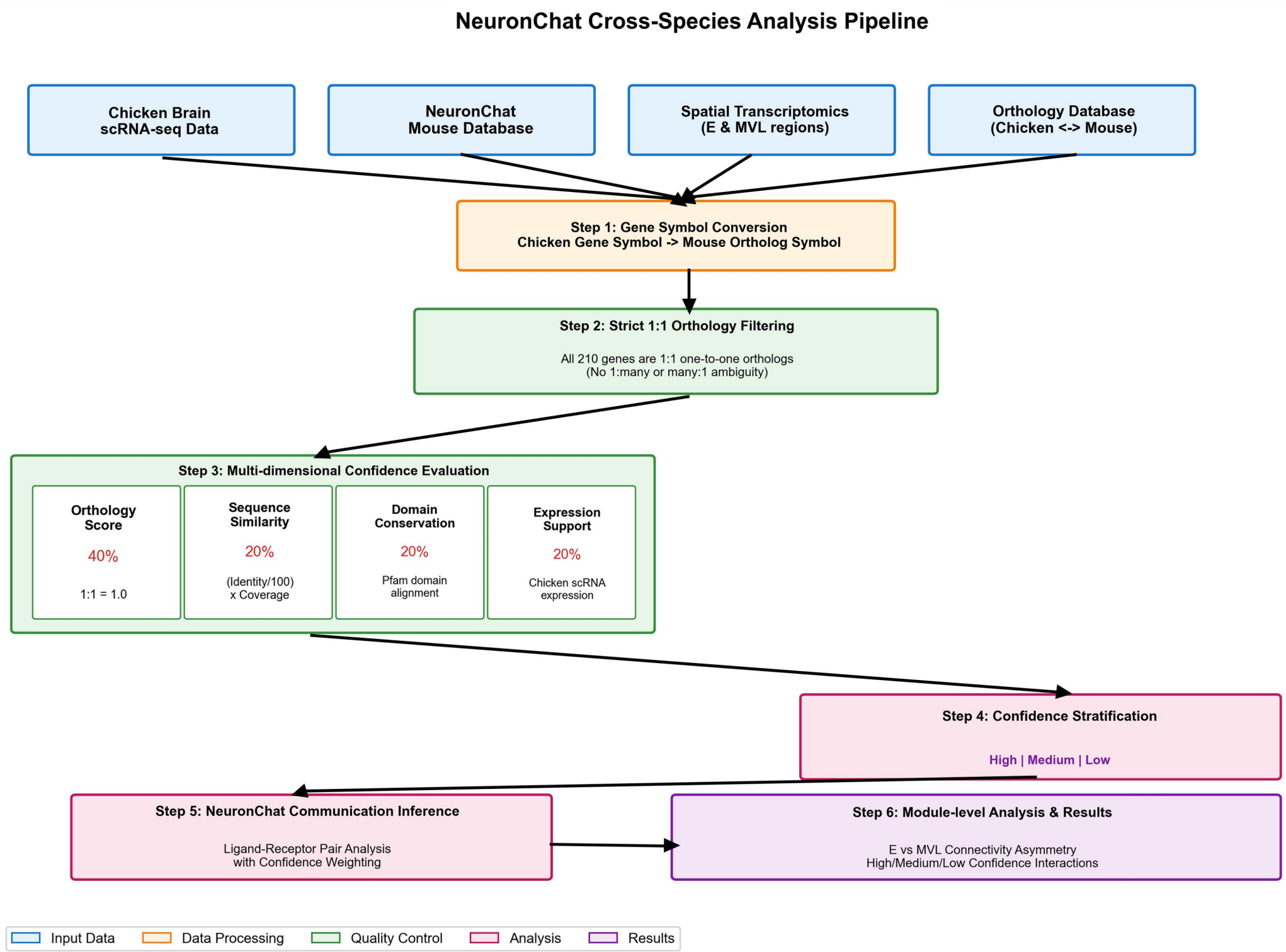

Figure S7. NeuronChat Cross-Species Analysis Pipeline

Related to Figure 3.

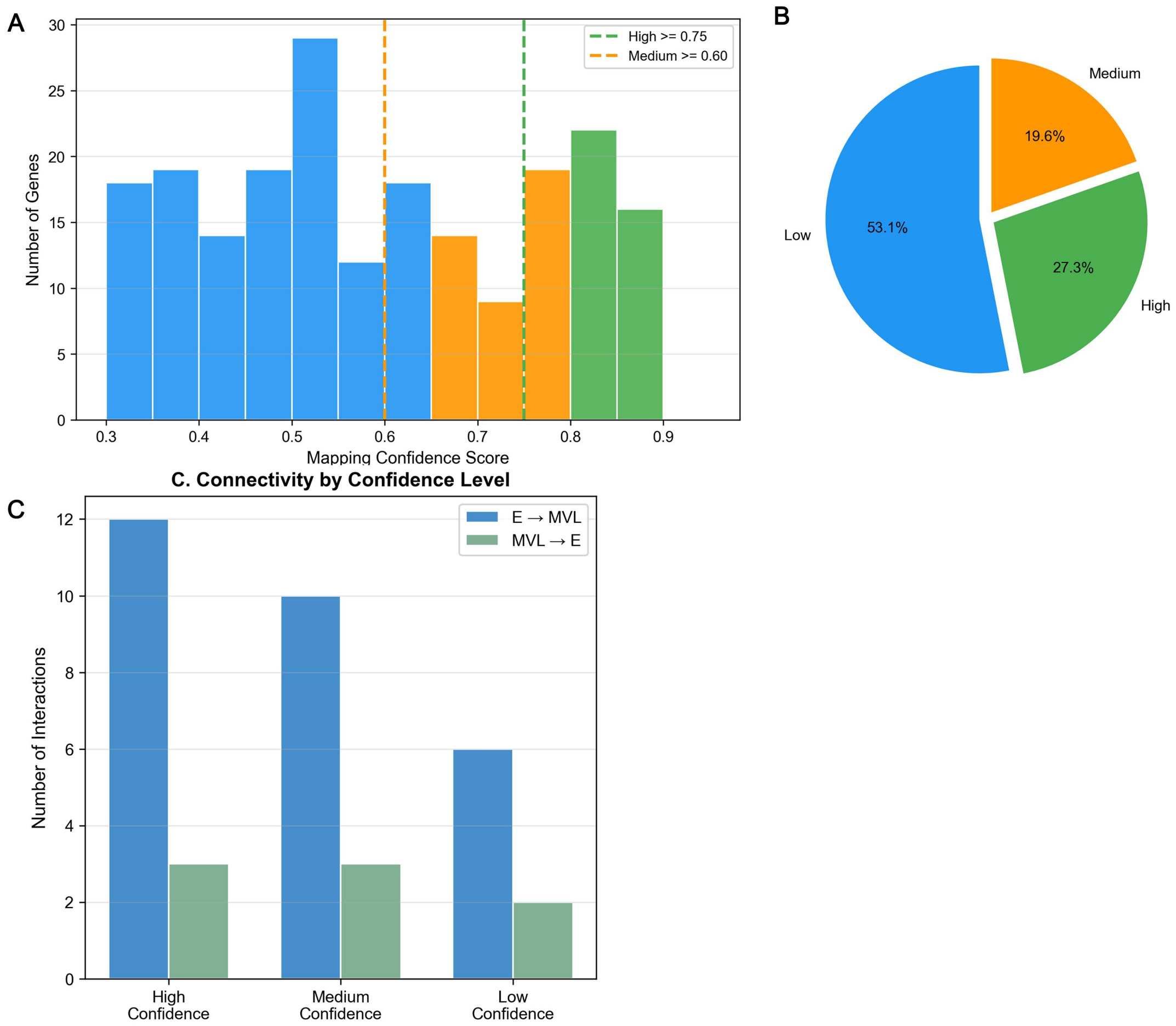

Figure S8. Mapping of Confidence Score and E-MVL Connectivity Asymmetry based on ligand-receptor interactions

Related to Figure 3.

(A) Confidence score. Histogram showing the confidence scores of 210 chicken-to-mouse orthologous genes. Dashed vertical lines indicate tier boundaries.

(B) Distribution of confidence levels. Pie chart illustrating the proportion of genes classified into each confidence tier.

(C) E-MVL connectivity asymmetry across confidence levels. Bar plots showing the number of ligand-receptor interaction pairs between E and MVL regions at different confidence tiers.
